# Pdgfrα^+^ stromal cells, a key regulator for tissue homeostasis and dysfunction in distinct organs

**DOI:** 10.1016/j.gendis.2024.101264

**Published:** 2024-03-09

**Authors:** Xia Kang, Kun Zhao, Zhu Huang, So-ichiro Fukada, Xiao-wei Qi, Hongming Miao

**Affiliations:** a Department of Pathophysiology, College of High Altitude Military Medicine, Army Medical University, Chongqing 400038, China; b Pancreatic Injury and Repair Key Laboratory of Sichuan Province, The General Hospital of Western Theater Command, Chengdu, Sichuan 610000, China; c Project for Muscle Stem Cell Biology, Graduate School of Pharmaceutical Sciences, Osaka University, Suita, Osaka 564-0871, Japan; d Department of Breast and Thyroid Surgery, Southwest Hospital, Army Medical University, Chongqing 400038, China; e Jinfeng Laboratory, Chongqing 401329, China

**Keywords:** Fatty infiltration, Fibrosis, Pdgfrα^+^stromal cell, Tissue degeneration, Tissue regeneration

## Abstract

Pdgfrα^+^ stromal cells are a group of cells specifically expressing Pdgfrα, which may be mentioned with distinct names in different tissues. Importantly, the findings from numerous studies suggest that these cells share exactly similar biomarkers and properties, show complex functions in regulating the microenvironment, and are critical to tissue regeneration, repair, and degeneration. Comparing the similarities and differences between distinct tissue-resident Pdgfrα^+^ stromal cells is helpful for us to more comprehensively and deeply understand the behaviors of these cells and to explore some common regulating mechanisms and therapeutical targets. In this review, we summarize previous and current findings on Pdgfrα^+^ stromal cells in various tissues and discuss the crosstalk between Pdgfrα^+^ stromal cells and microenvironment.

## Introduction

Extracellular matrix (ECM) remodeling occurs in tissue regeneration, repair, and degeneration. Proper ECM remodeling benefits for activation and migration of stem cells and facilitates tissue regeneration. On the other hand, excessive and uncontrolled ECM remodeling can cause fibrotic scar formation, fatty infiltration, and heterotopic ossification, leading to organ or tissue dysfunction.^1^, ^2^, ^3^, ^4^, ^5^

Mesenchymal stromal cells are indispensable for ECM remodeling and actively participate in maintaining tissue homeostasis. In the recent decade, a group of mesenchymal stromal cells specifically expressing platelet-derived growth factor receptor α (Pdgfrα) aroused researchers' interest. So far, Pdgfrα^+^ stromal cells in skeletal muscles were detailly studied and reviewed timely.^6^, ^7^, ^8^, ^9^ They present fascinating properties during muscle injury or atrophy. At first, muscle-resident Pdgfrα^+^ stromal cells were found to have a “double-edged sword” effect on muscle regeneration and degeneration. These cells are indispensable for the activation of muscle stem cells (MuSCs, also called satellite cells) during muscle regeneration. On the other hand, they accumulate in degenerative muscles and differentiate into myofibroblasts or adipocytes, leading to fibro-fatty infiltration, thus they are also called fibro-adipogenic progenitors (FAPs). In subsequent studies, muscle-resident Pdgfrα^+^ stromal cells are found not only to participate in ECM remodeling but also actively communicate with other cell types, especially immune cells and stem cells through direct and indirect ways. These findings indicated that muscle-resident Pdgfrα^+^ stromal cells exerted much more complex effects on regulating the microenvironment than expected. On the other hand, it has been well known that the fate of muscle-resident Pdgfrα^+^ stromal cells is determined by surroundings. These findings reveal the muscle-resident Pdgfrα^+^ stromal cells play a central role in maintaining muscle homeostasis.

Besides muscles, Pdgfrα^+^ stromal cells are subsequently identified in multiple organs and tissues, including artery, heart, pulmonary, tendon, bone, and adipose tissues. Although these tissue-resident Pdgfrα^+^ stromal cells have distinct names in these tissues, it is interesting that they share many common characteristics. All these groups of cells are involved in tissue fibrosis formation, and most of them have the adipogenic capacity or the ability to store lipids. In addition, these groups of cells participate in both tissue regeneration and degeneration and are indispensable for supporting the activation of stem cells. Finally, the biomarkers of these tissue-resident Pdgfrα^+^ stromal cells are similar.

In this review, we comprehensively introduce and discuss the properties of Pdgfrα^+^ stromal cells in distinct tissues and the crosstalk between these cells and the microenvironment in various conditions.

## The general properties of Pdgfrα^+^ stromal cells (FAPs) in skeletal muscles

Muscle-resident Pdgfrα^+^ stromal cells (also called FAPs) should be isolated using fluorescence activated cell sorter. Either Lin^−^ (lineage)^−^ Sca1^+^ cells or Lin^−^ Pdgfrα^+^ cells can refer to this group of stromal cells. Sca-1 can be used as a gating marker instead of Pdgfrα when sorting out muscle-resident FAPs,^10^ because 85% of muscle-resident Pdgfrα expressing cells were Lin^−^ Integrinα7^−^ Sca-1^+^.^11^^,^^12^ FAPs in muscle may also express CD29 (∼90.0%), CD34 (∼30.0%), and CD90 (∼60.0%), but these markers are not specific for FAPs. It has been reported that these markers are also highly expressed in satellite cells.^11^ Sca-1 cannot be used to define human FAPs since it is not expressed in human cells. Uezumi et al indicated that the CD56^−^ CD82^−^ CD318^−^ Pdgfrα^+^ CD201^+^ marker could be used to identify human muscle-derived FAPs.^13^ Furthermore, a recent study used CD34^+^ CD56^−^ CD45^−^ CD31^−^ as the strategy for isolating human FAPs (Table 1).^14^

**Table 1:** Biomarkers of Pdgfrα^+^ stromal cells in different tissues in human or mice.

Tissues	Species	Gating Strategy	Refs
Muscle	Mouse	Lin(−):SM/C-2.6(+):PDGFRα(+)	Uezumi et al, 2010^11^
		Lin(−):α7(−):Sca-1(+):CD34(+)	Joe et al, 2010^12^; Marcelin et al, 2020.^139^
		Lin(−):Ter119(−):α7(−):Sca-1(+):CD34(+)	Malecova et al, 2018.^15^
		Lin(−):α7(−):Sca-1(+)	Lemos et al, 2015^2^; Heredia et al, 2013^77^; Dong et al, 2014.^10^
		Lin(−):α7(−):PDGFRα(+)	Saito et al, 2020^45^; Dong et al, 2017.^95^
		Lin(−):Ter119(−):Sca-1(+)	Contreras et al, 2019 & 2020.^24^^,^^25^
		Lin(−):Podoplanin(+):PDGFRα(+)	Kuswanto et al, 2016.^81^
		Lin(−):PDGFRα(+)	Uezumi et al, 2011.^4^
	Human	CD56(−):CD82(−):CD318(−): PDGFRα(+):CD201(+)	Uezumi et al, 2016.^13^
		CD15(+):PDGFRα(+):CD56(−)	Arrighi et al, 2015.^31^
		CD34(+):CD56(−):CD45(−):CD31(−)	Farup et al, 2021.^14^
Adipose	Mouse	Lin(−):CD29(+):CD34(+):Sca-1(+)	Rodeheffer et al, 2008.^129^
		Lin(−):Gp38(+):PDGFRα(+)	Marcelin et al, 2017.^133^
		Lin(−):α7(−):Sca-1(+):CD34(+)	Lemos et al, 2012.^132^
	Human	Lin(−):CD34(+):CD44(+):PDGFRα(+)	Marcelin et al, 2017.^133^
Tendon	Mouse	TPPP3(−):Sca-1(+)PDGFRα(+)	Harvey et al, 2019.^46^
Lung	Mouse	Lin(−):Sca-1(+):CD34(+):Thy-1(+):PDGFRα(+)	McQualter et al, 2009.^149^
Heart	Mouse	Lin(−):Sca-1(+):PDGFRα(+)	Soliman et al, 2020.^147^
Bone marrow	Mouse	CD45(−):TER119(−):Sca-1(+):PDGFRα(+)	Houlihan et al, 2012^158^; Mashimo et al, 2019.^159^

In uninjured muscles, FAPs locate in the interstitial space of muscle tissue. Although FAPs are close to vessels, they are proven to be distinct from pericytes and vascular smooth muscle cells.^11^ In the context of muscle injury, FAPs undergo rapid proliferation, immigrate to injured sites, and rapidly accumulate circumferentially around injured muscle fibers. Interestingly, FAPs in glycerol-induced fatty degenerative muscle present with a round type, while they present with a typical elongated spindle shape in cardiotoxin-induced regenerative muscle.^11^ The reasons causing this difference in morphology are still unclear, and we speculate the fate of differentiation may be one underlying reason.

### Major subpopulations of muscle-resident FAPs and their properties

It has been identified that a single FAP possesses the ability to differentiate into a myofibroblast or an adipocyte in response to specific induction.^4^ However, heterogeneity of FAPs has been noticed recently. Subgroups of FAPs play different roles in muscle development and injury. Malecova et al divided FAPs into four subgroups based on the expression of vascular cell adhesion molecule 1 (Vcam1) and Tie2 (encoded by gene Tek), *i.e.*, Tie2^high^/Vcam1^−^, Tie2^low^/Vcam1^−^, Vcam1^+^, and double negative subgroup, of which Tie2 expresses low in Vcam1^+^ subgroup. Vcam1^+^ subgroup can only be observed in muscle injury, while Tie2^high^/Vcam1^−^ subgroup and Tie2^low^/Vcam1^−^ subgroup were likely present in all conditions. Moreover, the time to reach the peak of the Tie2^high^/Vcam1^−^ subgroup and the Tie2^low^/Vcam1^−^ subgroup was earlier and reduced more quickly than that of the Vcam1^+^ subgroup in the acute injury model. Interestingly, Vcam1^+^ FAPs appeared a high proliferative capacity and are closely associated with fibrotic phenotype in both acute muscle injury and muscle atrophy, implying this subgroup may be critical to fibrosis formation in muscles.^15^ Liu et al compared the role of Tie2^+^ progenitors and Pdgfrα^+^ progenitors in rotator cuff muscle injury using enhanced green fluorescent protein transgenic mice. They found that Pdgfrα^+^ progenitors are prone to adipogenesis while Tie2^+^ progenitors are enriched in biomarkers of myofibroblast.^16^ Because Tie2 is highly expressed both in FAPs and myofibroblasts,^11^ it could not be used as a specific marker for FAPs. However, Tie2^+^ could be a useful indicator for the fibrotic phenotype (Table 2). In another study, Giulio et al discovered that a subset of FAPs with higher expression of Sca-1 was more likely to differentiate into adipocytes.^17^

**Table 2:** The function of major subgroups of Pdgfrα^+^ stromal cells in varieties of diseases in each organ.

Tissues	Biomarkers for subgroups	Background	Features	Refs
Muscle	Vcam1(−)/Tie2^high^, Vcam1(−)/Tie2^low^ and Tie2^low^/Vcam1	Acute injury and dystrophy	Vcam1^(−)^/Tie2^high^ and Vcam1^(−)^/Tie2^low^ expanding immediately after acute injury, while Vcam1^(−)^/Tie2^high^ decrease firstly following by Vcam1^(−)^/Tie2^low^, the peak of Tie2^low^/Vcam1 is later than the other two groups. Vcam1^(−)^/Tie2^high^:muscle growth, Vcam1^(−)^/Tie2^low^:neo-angiogenesis, Tie2^low^/Vcam1: fibrosis	Malecova et al, 2018.^15^
	Wisp1(+), Dlk1(+), Osr1(+) Dpp4(+), Osr1(+) Cxcl14(+)	Regeneration	Wisp1(+) sub for ECM remodeling, increases at early stage after injury, Osr1(+) sub is the dominant subgroup at late stage	Oprescu et al, 2020.^19^
	CD90(+) and CD90(−)	Fatty infiltration	CD90(+) FAPs in human muscles are apt to adipogenesis	Farup et al, 2021.^14^
Adipose	CD9^low^ and CD9^high^	High fat diet	CD9^low^ sub representing adipogenic potential and CD9^high^ sub revealing a pro-fibrotic phenotype	Marcelin et al, 2017.^133^
	CD55 and IL13ra1, VAP1 and Adam12, CD142 and ABCG1	Adipogenesis	CD142 (+): ABCG1 (+) sub (Areg) showing an inhibitory effect on adipogenesis,	Schwalie et al, 2018.^130^
	DPP4(+)	Adipocyte development	highly proliferative and multipotent progenitors, which can give rise to ICAM1+ and CD142+ preadipocytes	Merrick et al, 2019.^134^
Tendon	TPPP3(+)Sca-1(−)Pdgfrα(+); TPPP3(−)Sca-1(+)Pdgfrα(+)	Tendon regeneration and scar formation	TPPP3+Sca-1-Pdgfrα+ subgroup is referred as tendon stem cells; TPPP3-Sca-1+Pdgfrα+ subgroup is tendon-resident FAPs, leading to scar formation after injury.	Harvey et al, 2019.^46^
Aorta	Sca-1(+)PDGFRα(+)PDGFRβ(−) and Sca-1(+)PDGFRα(+)PDGFRβ(+)	Aorta injury	Sca-1(+)PDGFRα(+)PDGFRβ(−) giving rise to smooth muscle cells in severe injury	Tang et al, 2020.^140^
Heart	Lin(−):CD29(+):mEF-SK4(+):PDGFRα(+):Sca1(+):periostin(+)	Heart failure	This subgroup secrets IL-17 to recruit immune cells and contributes to fibrosis	Chen et al, 2018.^144^

Since FAPs go through different stages during muscle injury, it is important to explore the dominant subgroup in each stage. Scott et al found that hypermethylated in cancer 1 (HIC1) enriched in mesenchymal progenitors (MPs) and regulated the MP quiescence. Depletion of HIC1 in MPs will cause impaired muscle regeneration. When analyzing the components of MPs, FAPs are found to be the major population. Then, the authors explored the trajectory of the proportion and the subgroups of HIC1^+^ MPs in injured muscles. The results showed that HIC1^+^ MPs reached the peak at the stage of injury, and returned to baseline at 28 days post-injury (DPI). Single-cell RNA-sequencing analysis showed that chemokines enriched in HIC1^+^ FAPs at 1 DPI, while cell proliferation associated biomarkers up-regulated at 2 DPI. After expansion, ECM proteins are highly expressed until 7 DPI, indicating an ECM remodeling process during muscle regeneration.^18^ Oprescu et al traced the dynamics and heterogeneity of whole FAPs during muscle regeneration using single-cell RNA sequencing. They found that FAPs and muscle fibers were two major cell groups in normal muscles. FAPs in injured muscles at an early stage (before 2 DPI) expressed abundant chemokines, including chemokine (C–X–C motif) ligand families and chemokine (C–C motif) ligand families, indicating that activated FAPs might have potential roles in regulating immune cells. Then, WNT1 inducible signaling pathway protein 1 (WISP1)^+^ FAPs increased at 3.5 and 5 DPI, and this subgroup enriched in fibrotic biomarkers. At 10 DPI, the regenerative stage of muscle repair, delta like non-canonical Notch ligand 1 (Dlk1)^+^ FAPs became a major group. Odd-skipped related transcription factor 1 (Osr1)^+^ FAPs were the dominant subgroup at 21 DPI.^19^ Those findings indicate that FAP subgroups are intimately regulated in different stages of muscle injury (Table 2). Another study showed that single-cell RNA sequencing of Pdgfrα^+^ cells in muscle identified six subpopulations, and the other five clusters can all be differentiated from a common Osr1^+^ cluster. Adam12^+^ cluster and Gap43^+^ cluster express the genes encoding the interleukin (IL)-4 receptor alpha and IL-13 receptor subunit alpha 1 with the ability to respond to IL-4 or IL-13 signal. Clu^+^ cluster is more likely to mineralization. Gli^+^ cluster and Hsd11b1^+^ cluster manifest an unparalleled neuromuscular junction association in response to nerve injury.^20^

According to research published by Hongchun Lin et al, FAPs from normal muscles could be divided into three subpopulations as C1, C2, and C3. However, in a mouse model of muscle atrophy caused by the denervation of the sciatic nerve, the three subpopulations from the denervated gastrocnemius muscle acted out transcriptional changes. Compared with normal FAPs, the denervated C1 subpopulation showed an apoptotic phenotype characterized by increased marks of apoptosis and the P53 pathway. Next, the denervated C2 subpopulation represented a pro-fibrotic phenotype with enriched denotes in epithelial–mesenchymal transition, transforming growth factor-β (TGF-β) signaling, and angiogenesis. Finally, the denervated C3 subpopulation unveiled pro-adipogenic features enriched with adipogenesis, MYC targets V1, and WNT/beta-catenin signaling.^21^

It was reported that diabetes mellitus could promote fibro-fatty infiltration in muscles. Farup et al analyzed the subgroups of FAPs in patients with diabetes. The findings suggested that CD90^+^ FAPs are associated with muscle degeneration under the regulation of PDGF signaling.^14^

### FAPs exhibiting multipotent capacity *in vitro* and *in vivo*

**Figure 1:**
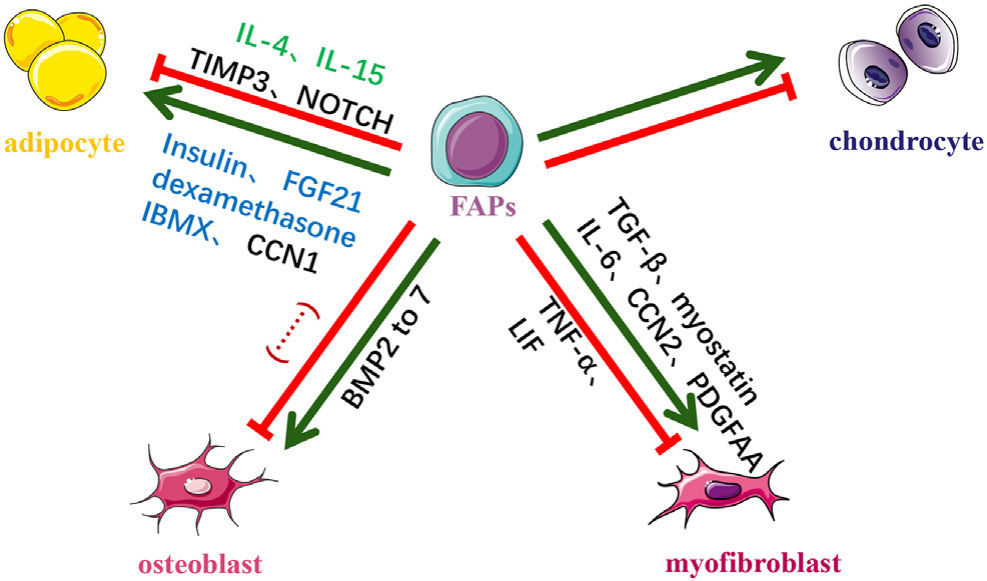
Multi-lineage differentiation of Pdgfrα^+^ stromal cells *in vitro* and *in vivo*. Pdgfrα^+^ stromal cells have multipotent capacity in different conditions. Representative signals regulating differentiation of Pdgfrα^+^ stromal cells *in vitro* and *in vivo* are listed in the figure; “green line” indicates promoted, “red line” indicates inhibited, and “…” represents no signals that have been identified currently.

**Figure 2:**
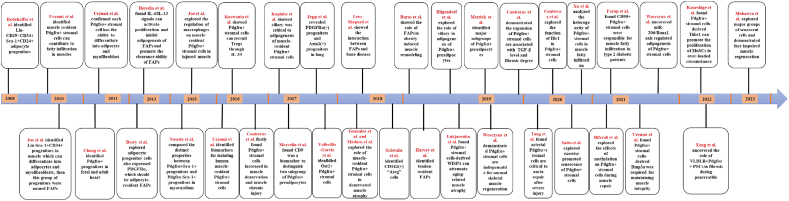
Representative milestone events leading to the discovery and development of Pdgfrα^+^ stromal cells in various tissues and diseases.

Muscle-resident FAPs may develop from embryonic interstitial muscle connective tissue cells.^22^ Uezumi et al and Joe et al examined the differentiation of FAPs and found that FAPs could commit to osteoblastic lineage, myofibroblasts, and adipocytes (Figure 1, Figure 2). Nevertheless, FAPs scarcely differentiate into myoblasts, indicating that FAPs belong to a lineage distinct from satellite cells.^11^^,^^12^ TGF-β and PDGF signaling are the two most important stimuli to commit FAPs to myofibroblasts. Treatment of TGF-β1 significantly enhances the expression of α-smooth muscle actin (α-SMA), the classic biomarker of myofibroblasts, as well as connective tissue growth factor (CCN2/CTGF), fibronectin, β1-integrin, and collagen I in FAPs.^2^^,^^11^^,^^12^^,^^23^, ^24^, ^25^ On the other hand, Pdgfrα regulates the fibrotic phenotype of FAPs. Mueller et al found intronic variants of Pdgfrα can be produced in FAPs with different polyadenylation sites, of which a protein isoform coded by one variant contained a truncated kinase domain. This isoform is highly expressed in regenerative FAPs and restrains the overactivation of Pdgfrα and attenuates fibrosis.^26^ Recent studies have confirmed that many skeletal muscles respond to the autotaxin/lysophosphatidic acid/lysophosphatidic acid receptors axis and trigger fibrosis. In mechanism, lysophosphatidic acid may scale up the number of FAPs in skeletal muscle through extracellular signal-regulated kinase 1/2 signal pathway, and promote their phenotypic differentiation into myofibroblast. After lysophosphatidic acid treatment, vinculin, vimentin, and α-SMA mRNA expression were up-regulated (markers of myofibroblast phenotype differentiation), while the protein and mRNA levels of adipoq decreased (adipocyte markers).^27^ Intracellular type II deiodinase and type III deiodinase may be associated with the differentiation of FAPs between adipocytes and fibroblasts. In a primary cell culture model, there were entirely different tracks of both enzymes through time. When FAPs differentiated forward adipocytes, type II deiodinase in FAPs reached the peak at the 50th hour and descend violently after that. Yet, type III deiodinase also climbed to the highest point as type II deiodinase began to decrease at the same time. When FAPs differentiated into myofibroblasts, type II deiodinase expression was at its highest point after two days, whereas type III deiodinase declined all the time to not de detected.^28^

Cocktails consisting of insulin, dexamethasone, and 3-isobutyl-1-methylxanthine can induce the adipogenesis of FAPs *in vitro*. Among the inducers in the cocktails, insulin is the most important adipogenic factor.^11^ In one study, IL-1β and IL-4 were used to induce M1 and M2 polarization of macrophages, respectively. IL-4-treated macrophages promoted while IL-1β-stimulated macrophages inhibited the adipogenesis of FAPs (Fig. 1).^29^ Preconditioning showed potential benefits in boosting muscle regeneration after ischemia-reperfusion injury. He Zhang et al authenticated that preconditioning stimulated FAPs differentiation into brown/beige-like adipocytes by modulating the β3AR signaling pathway, thereby expediting muscle regeneration proved by improvement of central nuclei regenerating myofibers after ischemia-reperfusion injury.^30^ Meanwhile, some other studies confirmed that FAP-derived adipose showed some features of brown adipose, which was sensitive to insulin-induced glucose uptake.^31^^,^^32^

**Figure 3:**
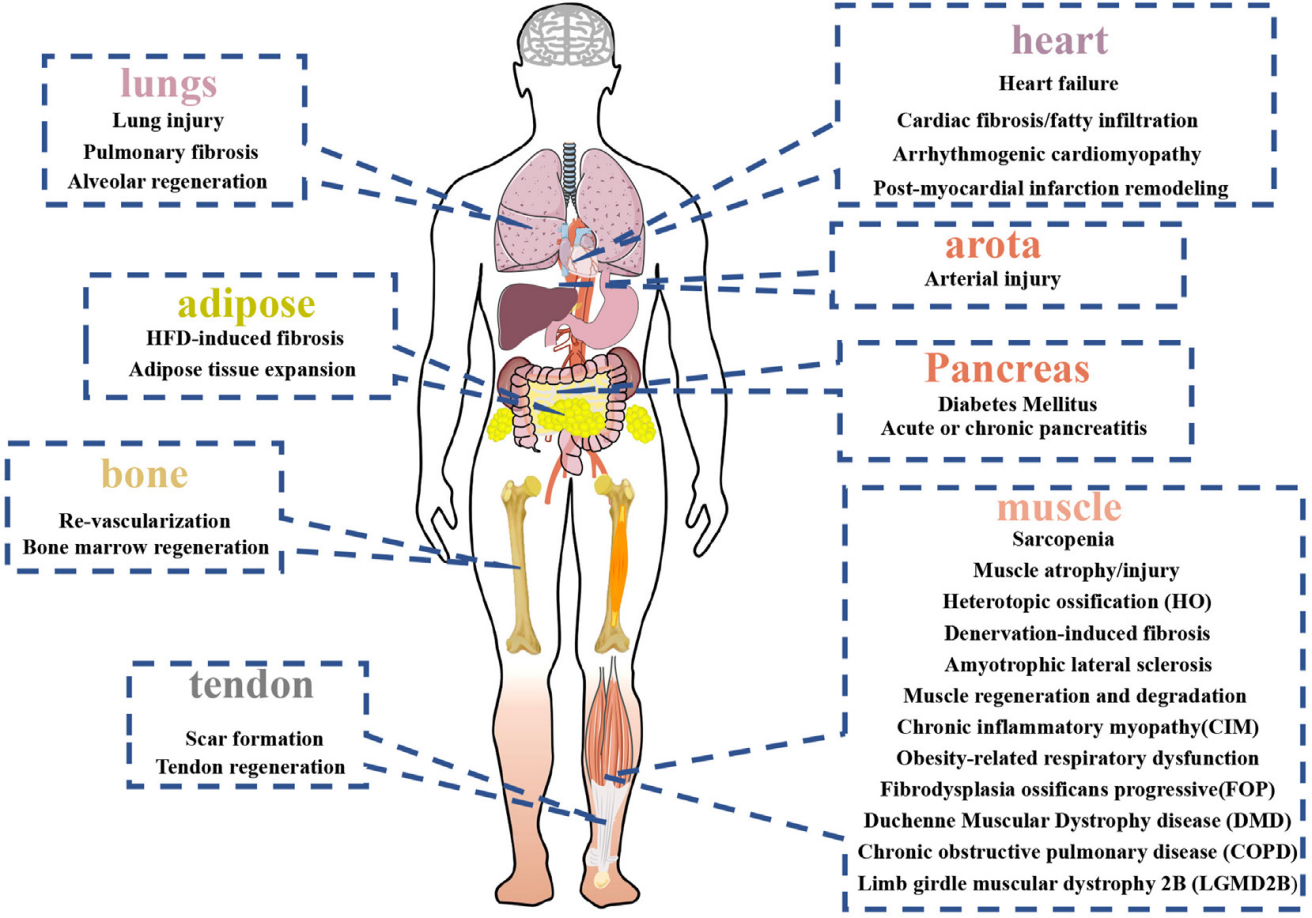
Biofunctions of Pdgfrα^+^ stromal cells in different diseases in various organs.

FAPs can commit to osteoblastic lineage. The member of bone morphogenetic protein (BMP) families can promote the osteoblastic induction of FAPs.^11^^,^^12^^,^^33^ They can lead to heterotopic ossification in some conditions. Oishi et al detected the osteogenesis capacity in human muscle progenitors and human FAPs. They found these two progenitors had similar differentiative capacities *in vitro*. However, only FAPs can successfully form a bone-like tissue *in vivo*. The skeletal muscle resident Tie2^+^ FAPs were the main initiator of heterotopic ossification in mice.^34^ Eisner and colleagues used BMP-2 treated acute injury model and demonstrated that FAPs, but not progenitors derived from circulation, were the main cellular source for heterotopic ossification. Inflammatory microenvironment perturbation may regulate the osteogenesis of FAPs in muscle injury.^35^ Furthermore, miRNA-146b-5p and miRNA-424 boosted the osteogenesis of FAPs.^36^ In addition to trauma, heterotopic ossification frequently occurs in nerve injuries. Sang et al revealed that calcitonin gene-related protein regulated heterotopic ossification after spinal cord injury (Fig. 3).^37^ In addition, IL-1 from activated monocytes can promote FAP mineralization demonstrated by up-regulated Runt-related transcription factor 2 expression in neurogenic heterotopic ossifications, which could be attenuated by supplementary anti-IL-1β neutralizing antibody.^38^ Moreover, FAPs contributed to hereditary heterotopic ossification disease, named fibrodysplasia ossificans progressiva. ACVR1 in FAPs could lead to heterotopic ossification phenotype in patients with fibrodysplasia ossificans progressiva after binding to its ligand, activin A,^39^ which we will discuss in a later section.

### The interaction between FAPs and microenvironment

FAPs have a “double-edged sword” effect during muscle regeneration and degeneration.^40^ Although FAPs are non-myogenic in nature, they can support the activation of muscle stem cells and facilitate muscle regeneration.^11^^,^^40^ On the contrary, FAPs accumulate excessively in degenerative microenvironment and then differentiate into adipocytes or myofibroblasts, thus promoting muscle atrophy.^24^^,^^41^^,^^42^ Unfortunately, it is impossible to prevent fatty infiltration and collagen deposition through diminishing FAPs, because ablation of FAPs can remarkedly impair muscle repair.^1^^,^^43^^,^^44^ In our opinion, generally, FAPs have a “repairman” role. In regeneration, they facilitate the muscle fiber formation followed by going back to a quiescent status to maintain muscle homeostasis. In degeneration, the continuously high level of FAPs might result from the compensatory response to activation failure of muscle stem cells, which are required to muscle repair. Subsequently, the increased FAPs might undergo adipogenesis or fibrogenesis to occupy the space which occurred after fiber atrophy.

In general, during muscle regeneration, FAPs undergo four important stages: proliferation, regeneration, senescence or apoptosis, and clearance and quiescence.^8^^,^^40^ However, the senescence or apoptosis process can be prevented in a degenerative environment. Then, FAPs may differentiate into adipocytes or myofibroblasts, thus leading to fatty degeneration.^45^ Timely apoptosis of FAPs is a key event for the switch between muscle regeneration and degeneration. The proliferation of FAPs at early stage benefits for activation of muscle stem cells, so inhibiting the proliferation of FAPs is not the best choice for preventing degenerative changes.^8^^,^^40^^,^^45^ Current strategies that prevent the progression of fibro-fatty infiltration and muscle degeneration often focus on inducing quiescence, senescence, or apoptosis to avoid abnormal accumulation of FAPs or preventing adipogenesis and fibrogenesis directly.

**Figure 4:**
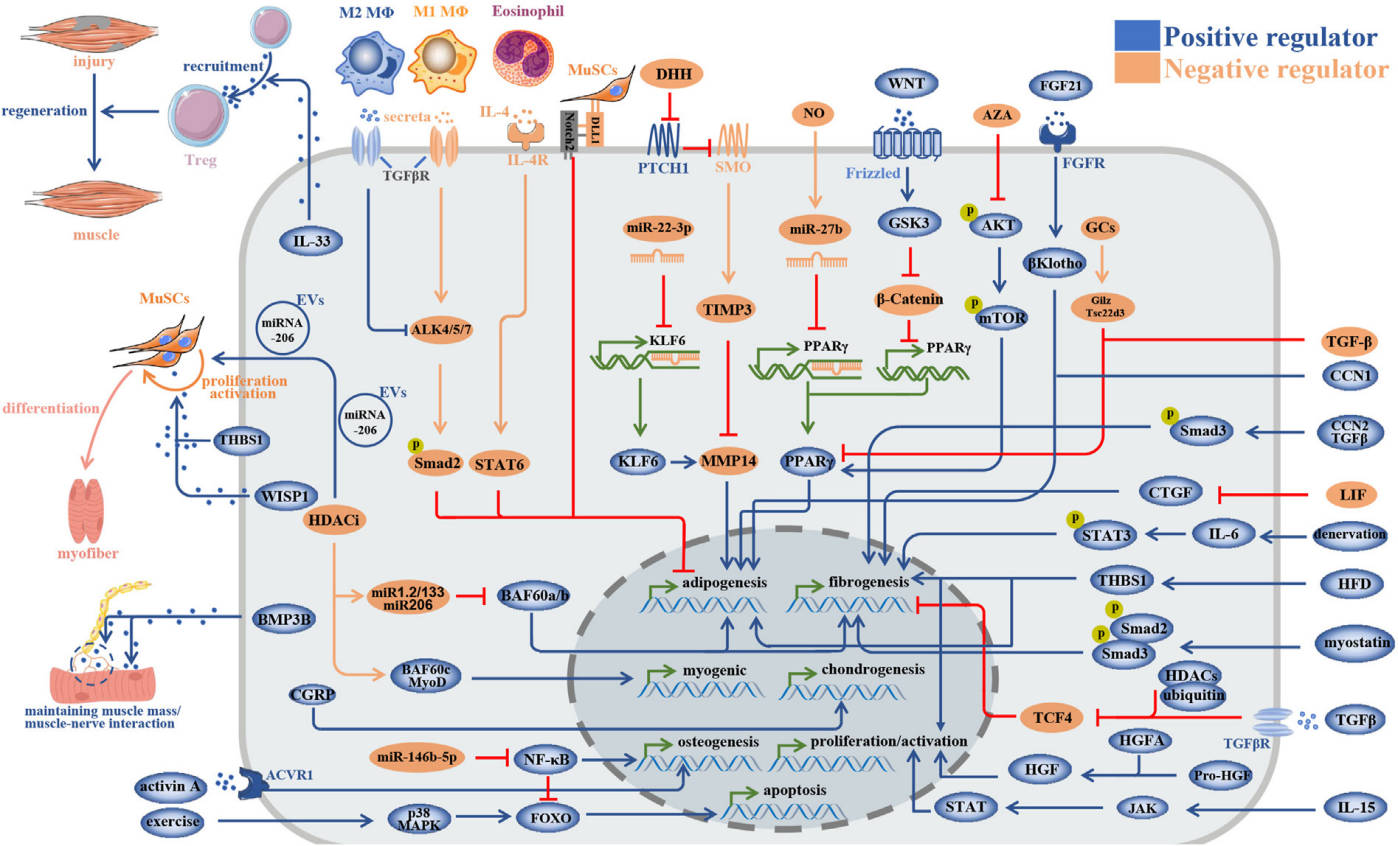
The classical pathways that regulate the communication between Pdgfrα^+^ stromal cells and microenvironment.

Since the crosstalk between FAPs and the microenvironment determined the fate of FAPs as well as remodeling the microenvironment, we hereby detailly describe the factors that play key roles in this communication (Fig. 4).

#### The role of growth factors in intercellular communications between FAPs and surroundings

Pdgfrα is an indispensable biomarker for the identification of FAPs, so PDGF ligands, especially PDGFAA, certainly play an important role in regulating FAPs. PDGFAA-Pdgfrα signaling contributes to the phenotypic switch toward pro-fibrotic FAPs.^7^^,^^46^ Intronic polyadenylation of Pdgfrα attenuates FAP activation and fibrosis.^26^ In addition, PDGFAA stimulates the TGF-β signaling through binding to Pdgfrα, thus promoting scar formation or fibrosis.^7^ Uezumi et al found that FAPs moderately expressed OB-Cadherin (Cadherin-11).^11^ It was reported that OB-Cadherin interacted with Pdgfrα.^47^ However, the effect of OB-Cadherin on FAPs is still unknown. It is interesting to explore the role of OB-Cadherin in regulating FAPs because OB-Cadherin regulates cell migration,^48^ differentiation, and angiogenesis.^49^, ^50^, ^51^, ^52^, ^53^, ^54^, ^55^

TGF-β is an important cytokine for FAP-mediated matrix remodeling and fibrosis. Unexpectedly, it can reduce the expression of Pdgfrα on FAPs. TGF-β promotes the myofibroblast differentiation, and then FAPs lose Pdgfrα expression during this process.^24^ The polarization of macrophages determines the production of TGF-β and survival of FAPs in injured muscles. TGF-β1 which is derived from Ly6C^low^ macrophages induces FAPs to a fibrogenic phenotype and results in collagen deposition in injured muscle. Furthermore, TGF-β can perturb TNF-α-induced FAP apoptosis in an adipogenic environment. Blockage of TGF-β by decorin can recover FAP apoptosis and reduce the fatty infiltration.^56^ TGF-β was found to down-regulate the expression of transcription factor 4 in FAPs through the ubiquitin-proteasome system and canonical Wnt/wingless signaling cascades.^25^ However, the activation of the TGF-β/Smad3 pathway in FAPs contributed to the fibrosis in amyotrophic lateral sclerosis-induced muscle atrophy.^57^ Recently, Uezumi et al identified BMP3B (GDF-10), a member of growth/differentiation factor families, was indispensable for maintaining muscle mass as well as muscle–nerve interaction. In sarcopenia, BMP3B expression was decreased in FAPs. Administration of ectogenic BMP3B efficiently reversed the aging-related muscle atrophy.^44^

The members of the fibroblast growth factor (FGF) family are critical to regulate fibrogenesis and adipogenesis of FAPs. Four FGF receptors (FGFR) can be identified in satellite cells and FAPs. FGF21-FGFR2-betaKlotho pathway promoted adipogenesis of FAPs.^58^ The elevated miR-214-3p had the potential to hasten FAP fibrogenesis by adjusting the FGF2/FGFR1/TGF-β axis, which shed light on new strategies for the treatment of fibrous degeneration of Duchenne muscular dystrophy (DMD) by interfering miR-214-3p.^59^ A recent literature by Sebastian et al demonstrated that more aberrant FGF-2-dependent signaling promoted the formation of intramuscular adipose tissue in skeletal muscle donated by older people who are more than 75 years old when compared with that in people who are less than 55 years old. Mechanically, the elevated FGF-2 in aged skeletal muscle cells irritated the adipogenic differentiation of FAPs through the FGF-2/FGFR/FRA-1/miR-29a/SPARC axis.^60^

Cellular communication network (CCN) factor families play a critical role in crosstalk between FAPs and surroundings. FAPs derived from young mice exhibit a higher potency in proliferation and adipogenesis but a lower ability for fibrotic differentiation when compared with those derived from elder mice.^61^ Young FAPs can significantly activate muscle stem cells through WISP1, which is also called CCN4. Injection of WISP1 can improve the muscle regeneration.^61^ Moreover, another study found that CTGF-CCN2 signals could promote denervation-induced fibrosis through a TGF-β-independent way.^23^ In addition, it is well known that chronic kidney disease can bring about fatty infiltration in obese people. ECM protein CCN1 is up-regulated in chronic kidney disease and commits FAPs to an adipogenic fate.^62^

It is still not fully understood how FAPs are activated after muscle injury. Joseph et al identified that the proteolytic process of hepatocyte growth factor could be activated after muscle injury. Hepatocyte growth factor activator can stimulate both FAPs and satellite cells to GAlert status for accelerating stem cell activation and tissue repair.^63^ In addition, HIC1 may play an important role in quiescence–activation transition in FAPs. Deleting HIC1 can arouse FAPs and impair the muscle regeneration.^18^^,^^64^

#### The function of transcriptional factors in determining the fate of FAPs

Currently, several transcriptional factors have been explored to regulate the proliferation and differentiation of FAPs. The key role of Osr1 in FAPs and the function of the Osr1^+^ FAP subgroup in muscle development and muscle injury have been increasingly emphasized. Osr1 can regulate the proliferation, apoptosis, adipogenesis, and quiescence of FAPs in injured muscles.^65^^,^^66^ Two subgroups of Osr1^+^ FAPs, which additionally express dipeptidyl peptidase-4 or C–X–C motif chemokine ligand 14, can be found in uninjured muscles.^19^ Another study identifies that Osr1 remains at a low level and frequency in adult FAPs. However, the expression of Osr1 was reactivated in FAPs after acute muscle injury. Osr1^+^ FAPs presented with an active phenotype.^65^ Interestingly, Osr1^+^ muscle connective tissue cells may be embryonic FAPs. During muscle development, these cells can partially give rise to adult FAPs and are critical to provide a pro-myogenic niche for myogenic progenitors. Osr1 deficiency leads to limb muscle patterning defects.^22^ These findings demonstrate that the Osr1^+^ subgroup of FAPs may be more primitive and active than Osr1^−^ FAPs.

Krüppel-like factor families were reported to regulate the differentiation of FAPs. Contreras et al explored that the bulk protein level of transcription factor 4/TCF7L2 was up-regulated due to the expansion of transcription factor 4-positive FAPs in dystrophic muscles, denervated muscles, and chronically damaged muscles.^67^ However, at the single-cell level, TGF-β-TGFBR1 signaling repressed the expression of transcription factor 4/Tcf7l2 through histone deacetylase (HDAC)-mediated degradation in FAPs and promoted fibrogenic differentiation.^25^ Krüppel-like factor 6 was reported to regulate the expression of matrix metalloproteinase 14,^68^ a critical factor to positively regulate adipogenesis of FAPs,^69^^,^^70^ targeting Krüppel-like factor 6 using miR-22-3p could efficiently down-regulate the expression of matrix metalloproteinase 14.^68^

Peroxisome proliferator-activated receptor γ (PPARγ) is a key regulator in adipogenesis. TGF-β1 and nitric oxide could inhibit the expression of PPARγ by up-regulating miRNA-27b, to prevent adipogenesis of FAPs.^24^^,^^71^ Furthermore, an immunosuppressant, named azathioprine, can negatively regulate the adipogenesis of FAPs by inhibiting the expression of PPARγ by inactivating the AKT-mTOR pathway.^72^ Recently, glucocorticoid chemical components were found to inhibit the adipogenesis of FAPs. They can induce the transcription of Gliz/Tsc22d3 and inhibit the expression of PPARγ.^73^ In addition, using a GSK3 inhibitor, LY2090314, can inhibit adipogenesis of FAPs through repressing PPARγ by inhibiting WNT/GSK/beta-catenin pathway.^74^ These studies together demonstrated that PPARγ should be a critical therapeutic target for adipogenesis of FAPs.

Microenergy acoustic pulses induced FAP brown/beige adipogenesis *in vitro* featured by induction of uncoupling protein 1, a hallmark of brown/beige fat, which could further secrete several growth factors accelerating muscle repair evidenced by other literatures.^75^

In one recent study, Wosczyna et al uncovered that the miR-206/Runt-related transcription factor 1 axis played an important role in regulating the adipogenesis of FAPs,^76^ demonstrating that miRNAs participated in regulating the fate of FAPs, which should be further investigated.

#### The role of inflammatory cytokines in intercellular communications between FAPs and surroundings

Inflammation takes place at the onset of muscle injury. The interaction between immune cells and FAPs is a key event in the early stage of acute muscle injury.^8^ The immune cells and FAPs could regulate each other through an indirect way.^8^^,^^19^

Eosinophils seem to play an opposite role in acute and chronic injured muscles. In glycerol-induced acute muscle injury, type 2 innate signals derived from eosinophils, *i.e.*, IL-4/IL-13 signaling, regulate FAPs to facilitate muscle regeneration. IL-4 can promote the proliferation of FAPs by activating the IL-4 receptor/STAT6 pathway and can inhibit adipogenesis of FAPs, thus preventing fatty infiltration in muscle injury.^77^ Moreover, IL-4 can enhance phagocytosis of FAPs. The phagocytic ability of endothelial cells, muscle progenitors (satellite cells), macrophages, and FAPs has been compared. Interestingly, FAPs are more efficient in phagocytizing necrotic debris compared with the other three cell types. Thus, IL-4 not only commits FAPs to regenerative fate in muscle injury but also promotes the clearance of necrotic debris.^77^ In another study, the researchers found glucocorticoid could induce adipose accumulation in muscle injury. However, treatment with IL-4 antagonizes this effect through IL4 receptors.^10^ These two studies together indicate that IL-4/IL-4 receptor signaling is critical to creating a regenerative environment by regulating the proliferation, adipogenesis, and phagocytosis of FAPs. However, Kastenschmidt et al stated that the elevation of eosinophils through a group 2 innate lymphoid cells (ILC2s)-derived IL-5 dependent way. The expanded ILC2s and eosinophils could negatively impact muscle regeneration and promote fibrosis in DMD.^78^ These findings implied that the function of eosinophils on muscle regeneration and degeneration is dependent on different circumstances.

Type І inflammatory cytokines prevent the adipogenic differentiation of FAPs. It was observed that IL-1α and IL-1β intensely restrained FAP adipogenesis. On the other hand, betacellulin and epidermal growth factor conspicuously facilitate FAP proliferation.^79^ TNF-α is a key cytokine that promotes apoptosis of FAPs in regenerative muscle. Ly6C^high^ macrophages induce FAP apoptosis by producing TNF-α and prevent collagen deposition. Contrarily, TNF-α may exacerbate fibrosis in degenerative muscles. Anti-TNF treatment can attenuate fibrosis in degenerative muscles.^2^ Mechanistically, TNF-α-stimulated fibrosis might be mediated partly through WNT/beta-catenin signaling.^25^

FAPs can also produce and secrete inflammatory cytokines to regulate other cells and influence muscle repair. Sarcopenia is commonly seen in the elderly, characterized by muscle wasting, fatty infiltration, and fibrosis.^80^ Impaired recruitment of regulatory T cells is an important reason for muscle regeneration failure in sarcopenia. Regulatory T cells marked by Foxp3 and CD4 are critical to tissue repair and regeneration. It was reported regulatory T cells significantly decreased in elder mice and the muscle regeneration was impaired.^81^ Kuswanto et al found that IL-33 was indispensable for maintaining the regulatory T cell homeostasis after muscle injury. Furthermore, they proved that FAPs were the dominant source of IL-33 in muscles. Muscle injury can lead to a mass of dead FAPs that can release abundant IL-33. Then, regulatory T cells can be recruited through IL-33/suppression of tumorigenicity 2 signal to injured muscle and facilitate muscle regeneration.^81^ Interestingly, in the study performed by Kastenschmidt et al which we discussed above, the expanded ILC2s and eosinophils were just regulated by IL-33 secreted by FAPs.^78^

Denervation-associated muscle atrophy is a common complication after nerve system injury or diseases. Several studies proved that FAPs expand in denervated muscles.^57^^,^^67^^,^^82^ Although denervation is less inflammatory muscle atrophy compared with acute muscle injury or inflammatory muscle diseases, Madaro et al revealed that denervation causes FAPs to secrete IL-6 through activation of the STAT3 pathway. Blockage of FAP-derived IL-6 can counter muscle atrophy and fibrosis in denervated muscles.^82^

#### Chromatin remodeling is critical to the cellular behavior of FAPs

Emerging evidence revealed that epigenetic remodeling of histones can change the natural fate of FAPs and regulate them to commit to myogenic phenotype. Noticeably, the pharmacological treatment with HDAC inhibitor (HDACi) has been under clinical trials on DMD. HDACi can induce FAPs to gain promyogenic phenotype in a dystrophic muscle environment at an early stage of DMD.^83^

HDAC/myomiRNAs/BAF60 axis is critical to this transition. The expression of myomiRNAs, such as miRNA-1.2, miRNA-133, and miRNA-206, up-regulate in FAPs after down-regulation of HDAC. These miRNAs favor the formation of BAF60C by targeting BAF60A and B. Furthermore, one study found that treatment with HDACi could promote the expression of miRNA-206 in extracellular vesicles, which promotes MuSC activation and muscle regeneration.^84^ However, current studies also demonstrated that FAPs derived from late-stage of DMD are resistant to HDACi-induced chromatin remodeling.^83^^,^^85^^,^^86^ Consalvi et al recently revealed the possible underlying mechanism. In the late stage of DMD, FAPs exhibit an aberrant HDAC activity which leads to pan hypo-acetylation at the promoters of genes regulating the cell cycle. This process cannot be fully reversed by HDACi. On the other hand, the author found that H3K9/14 hyper-acetylation at promoters of senescence associated secretory phenotype genes in natural FAPs at a later stage of DMD could be inhibited by HDACi and the fibrosis can be attenuated.^87^ In addition, several studies confirmed in recent years that HDACi could attenuate fibrosis in multiple organs and tissues.^88^^,^^89^ For muscles, using HDACi could attenuate the fibro-adipogenic phenotype of FAPs.^84^^,^^90^

Besides acetylation, methylation regulated the myogenic fate of FAPs. The cooperation between PR domain-containing 16 and G9a/GLP (H3K9 methyltransferases) could silence myogenic genes of FAPs, thus repressing their myogenic fate, which was evidenced by enriched H3K9me2 levels of regulatory genomic loci of myogenic genes (MyoD transcriptional start site, TSS; MyoD core enhancer, CE; Desmin, Des).^91^

#### The myokines regulating the fate of FAPs

During muscle retraction, myofibers can produce a set of active factors called myokines in paracrine- and autocrine-dependent manners.^92^ These myokines accumulate locally to form a special “myokine microenvironment”, which plays a critical role in regulating physiological and pathological processes.

Limited studies explored how myokines affect FAPs. In our previous study, we tested the levels of several myokines in muscle injury compared with those in normal muscles. The results showed that IL-15 might participate in regulating the biological behavior of FAPs. Further investigation revealed that IL-15 can stimulate the proliferation of FAPs through JAK/STAT pathway. Moreover, treatment with IL-15 can prevent fatty infiltration and promote muscle regeneration.^93^ In another study, Steven and colleagues found that overexpression of leukemia inhibitory factor, another myokine, could suppress FAPs and attenuate fibrosis through abrogating TGF-β signaling.^94^

Interestingly, myokines may also participate in muscle degeneration caused by systematic disease. A recent report showed that myostatin exacerbated collagen deposition in muscles in chronic kidney failure. Overexpression of myostatin promoted FAPs to differentiate into myofibroblasts through activation of Smad3 signaling.^95^

#### The role of FAP senescence in muscle regeneration

Senescent cells could permanently go into cell-cycle arrest and secrete abundant cytokines called senescence associated secretory phenotype. Cellular senescence can not only occur in older individuals but can be found in whole life. In recent years, the effects of senescent FAPs on muscle regeneration and degeneration were detailly studied.

Recently, Emily Parker et al decoded that FAPs-derived extracellular vesicles presented a significant ascension in miRNAs, such as miR-124, miR-181a, miR-let-7b, and miR-let-7c after 14 days’ single-hindlimb immobilization in mice, which have formerly been testified to play vital roles in cellular senescence and muscle atrophy. However, the ascending effect of miRNAs was not significantly changed in FAP-derived extracellular vesicles from the IL-1β knockout mice miraculously. These data supported the idea that IL-1β activated by muscle disuse could directly stimulate the liberation of atrophy and senescence associated miRNAs.^96^

On the other hand, adenosine 5′-monophosphate (AMP)-activated protein kinase (AMPK) pathway is considered to be an important regulator for FAP senescence. Saito et al compared the senescence of FAPs between muscle acute injury and idiopathic inflammatory myopathies. The authors found that exercise could induce the senescent phenotype of FAPs and promote muscle regeneration. The astriction of exercise on FAPs also was observed by Valero et al.^97^ However, the senescence of FAPs regulated by exercise was prevented in chronic myopathy. The AMPK pathway is a possible regulator that makes this discrepancy. Administering AICAR, an agonist of the AMPK pathway, could restore the senescent phenotype of FAPs and improve muscle function.^45^ Consistently, Liu et al also found inhibiting AMPK in FAPs enhanced the expression of p65 and TGF-β1 and induced an apoptosis-resistance phenotype. Furthermore, conditional depletion of AMPKα1 in FAPs enhanced the fibrogenic phenotype of these cells.^98^

On the contrary, some other important studies found senescent FAPs exerted a harmful effect on muscle regeneration. Using Z24^−/−^ mice, an accelerated aging mouse model, Liu et al found that nearly half of FAPs presented with a senescent phenotype, the proportion was much higher than that in wild-type muscles. These senescent FAPs could inhibit the proliferation and differentiation of MuSCs. Administration of senolytics could efficiently transfer the senescent FAPs to apoptotic phenotype and restore the number of MuSCs.^99^ Another study recently published by *Nature* resolved an atlas of senescent cells in muscles. The authors first established a technology that could identify and isolate senescent cells *in vivo*. They found senescent cells existed in both cardiotoxin-injured muscles from young and old mice. Macrophages, FAPs, and MuSCs were three major groups in senescent cells. Clearance of senescent cells could improve muscle regeneration and attenuate fibrosis.^100^

The paradox about the function of senescent FAPs on muscle regeneration is still unclear. The differences in disease models or interventions may be possible reasons to explain the discrepancy. However, the detailed underlying mechanism should further be explored and verified.

Another aspect should be noticed is that the relationship between senescence and apoptosis. Although senescence and apoptosis are two distinct cellular processes, they share the same pathway and stimuli partially. Many genes can be involved in both senescence and apoptosis, such as TRP53. Furthermore, varieties of senescent inducers may have the ability to induce apoptosis based on the different doses. One therapeutical strategy to treat cellular senescence is inducing the senescent cells into apoptosis. Thus, we think senescence and apoptosis may often coexist in complex circumstances, such as injured or atrophied muscles.

#### Cilia regulating the adipogenesis of FAPs

Previous studies demonstrated that cilia were important to FAPs. Kopinke et al found that fatty degeneration was largely prevented after removing cilia from FAPs. Desert hedgehog protein secreted by Schwan's cells could inhibit the expression of PTCH1 and activate Smoothened on cilia, thus promoting the production of tissue inhibitor of metalloproteinase 3. The overexpression of tissue inhibitor of metalloproteinase 3 restricted the adipogenesis of FAPs through a non-cell-autonomous mechanism, which dominantly reversed the adipogenic induction of matrix metalloproteinase 14.^33^ Yamakawa et al found that ablation of trichoplein keratin filament binding gene could induce ciliary elongation on FAPs in injured muscles, stimulate muscle regeneration, and inhibit adipogenesis. Mechanically, insulin/Akt and IL-33/suppression of tumorigenicity 2/JNK pathways regulated the dysfunction of cilia-dependent lipid raft dynamics and the expression of IL-13, which facilitated myoblast proliferation and M2 macrophage polarization.^101^ Yao et al defined a subpopulation of muscle-resident FAPs characterized by heightened Hedgehog signaling, namely Gli1^+^ FAPs. Gli1^+^ FAPs with elevated heightened Hedgehog signal promoted regeneration of skeletal muscle by delivering trophic signals to support myogenesis while placing restrictions on adipogenic differentiation.^102^

#### Obesity and HFD significantly impacting the fate of FAPs

It is well known that FAPs and myocytes are overloaded with lipids in obesity.^103^ Obesity commonly results from a long-term high-fat diet (HFD). In mouse models, HFD can regulate the proliferation of intradiaphragmatic FAPs through up-regulating serum thrombospondin 1. HFD can also promote the adipogenesis and fibrogenesis of FAPs. Those pathological changes of FAPs lead to diaphragm contractile deficits and finally induce respiratory dysfunction.^104^ However, it was reported that short-term HFD could reprogram mitochondrial dysfunctions by regulating the beta-catenin/follistatin axis, thus ameliorating the pathological changes in dystrophic mice.^105^

Mogi et al investigated the intramuscular fat deposition in wild-type mice and several diabetic mouse models, including KKAy mice, db/db mice, and diet-induced diabetic mice. They found that diabetes promoted aging-related obesity by inducing aberrant adipogenesis of FAPs.^106^ Souza et al studied the effects of obesity and exercise on muscles exposed to radiation, and they found that HFD could increase fibrosis and fatty infiltration in muscles. Moreover, they noticed that the infiltration of FAPs in muscle was reduced in obese mice with treadmill exercise.^107^ Sergio Perez-Díaz et al discovered that mice skeletal muscle FAPs could highly secrete nidogen-1 in response to HFD. Upward nidogen-1 from FAPs eroded the proliferation of muscle stem cells and triggered the fibrogenic fate of FAPs and sacrificed their adipogenic potential, accounting for an overaccumulation of ECM.^108^

As described before, Farup et al analyzed the subgroup of FAPs in patients with type 2 diabetes mellitus. The authors isolated CD90^+^ FAPs as the major group to be responsible for fatty infiltration. PDGF was critical to induce CD90^+^ FAP proliferation and ECM remodeling during fatty infiltration. Treatment with metformin could reduce this process.^14^

#### Interaction between FAPs and MuSCs

The communications between FAPs and MuSCs are core events during muscle regeneration and degeneration. They can regulate each other through a direct or indirect way. Uezumi et al unveiled that satellite cells and myotubes could inhibit the adipogenesis of FAPs. Further investigation indicated that it worked through direct cell–cell contact.^11^ The mechanism of this interesting finding was explained in the following study. NOTCH signals were proven to be critical to this process. Delta-like canonical Notch ligand 1, the Notch ligand expressing on myofibers and muscle stem cells, was able to activate NOTCH signaling in FAPs through direct cell–cell contact and prevented adipogenesis. However, in a degenerative environment, FAPs were insensitive to NOTCH signals, which resulted in the accumulation of adipocytes.^109^ In another study, Moratal et al co-cultured human FAPs with myogenic progenitors and found that myogenic progenitors modulated FAPs through soluble factors. Activation of the PI3K-AKT pathway in MuSCs promoted the proliferation of FAPs. In addition, MuSCs regulate adipogenesis and fibrogenesis of FAPs through phosphorylation of Smad2 and up-regulation of Gli1. However, this aforementioned mechanism did not exist in FAPs and myogenic progenitors derived from aged people or patients diagnosed with DMD.^110^ In addition, some studies showed muscle contraction could impact fatty infiltration. It was reported that short-term limb disuse could lead to fatty infiltration.^111^ However, another study reported that limb unloading could prevent fatty infiltration and muscle degeneration in injured muscles.^112^ The detailed underlying mechanism should be further investigated.

On the other hand, numerous studies demonstrated that FAPs could regulate MuSCs by secreting varieties of cytokines positively or negatively. Mechanical stress can change the secreta of FAPs to facilitate myoblast activation.^113^ In an over-loaded model, the expression of thrombospondin 1 in FAPs was enhanced under the regulation of Yap/Taz. The secreted thrombospondin 1 can promote the proliferation of MuSCs through binding to its receptor CD47.^114^ Schuler et al identified that FAPs excreted SPARC related modular calcium binding 2 protein which could accrue with aging. Rising SPARC related modular calcium binding 2 conduced to the deformed integrin beta-1/mitogen-activated protein kinase signaling during aging, thus resulting in damaged MuSC functionality and muscle regeneration.^115^

In other studies, the frequencies of FAPs in denervated muscles or chronic inflammatory myopathy were also much higher than those in acutely injured muscles. Senescent FAPs expressed higher levels of IL-33 and TGF-α-stimulated gene-6. These two cytokines participated in muscle regeneration by regulating macrophages and regulatory T cells. In addition, the “don't eat me” signals CD274 and CD47 were up-regulated in aberrant activated FAPs, indicating that FAPs in degenerative muscles could not be efficiently cleared.^45^

In sarcopenia, the secretome of FAPs was significantly changed and lost their ability to support muscle homeostasis. Lukjanenko et al compared the properties of FAPs in young and old mice. They found FAPs showed a tendency to fibrogenesis but with less capacity for adipogenesis. Moreover, the authors demonstrated that FAPs isolated from old mice showed an impaired ability to activate MuSCs. Mechanically, WISP1 decreased in FAPs from old mice. Administration of WISP1 could efficiently restore the myogenic capacity of MuSCs and promote muscle regeneration.^61^ Moreover, denervation at neuromuscular junctions can be identified in sarcopenia. Uezumi et al found Bmp3b expressed by FAPs was critical to maintaining the myofiber mass and muscle–nerve interaction. However, the level of Bmp3b decreased in sarcopenia.^44^

#### ACVR1 is critical to the osteogenesis of FAPs

Activin A-ACVR1 axis is a critical pathway regulating osteogenesis of FAPs. Activin A excessively triggers canonical BMP signaling in FAPs through mutated ACVR1 receptors, which exacerbates the deterioration of fibrodysplasia ossificans progressiva. However, an artificial antibody (JAB0505) against the extracellular domain of ACVR1 aggravated heterotopic ossification unexpectedly. This raised a concern for the safety and effectiveness of anti-ACVR1 antibodies.^116^ Overexpression of ACVR1 in Tie2^+^ FAPs prevented fibrodysplasia ossificans progressiva mice from heterotopic ossification induced by injury by seizing a seat for essential signaling components and by conversing ACVR1 (R206H) into inactive or less active receptor complexes.^117^ In addition, treatment with palovarotene was able to efficiently reduce the abnormal expansion of FAPs in fibrodysplasia ossificans progressiva and finally attenuated heterotopic ossification progression.^118^

Besides to fibrodysplasia ossificans progressiva, Stanley et al explored that overexpression of ACVR1R^206H^ negatively influenced muscle regeneration after injury. The myogenic potential of MuSCs in ACVR1^R206H^ knock-in mice was impaired. FAPs from ACVR1^R206H/+^ mice repressed myotube formation.^119^

### FAPs as a promising potential therapeutical target

Several studies tried to treat muscle atrophy by targeting FAPs. Fiore and colleagues treated muscle fibrosis by using Nilotinib, a tyrosine kinase inhibitor. Unfortunately, muscle regeneration was simultaneously impaired when fibrosis was prevented.^1^ The reason may be that nilotinib also disrupts myogenic progenitor differentiation.^120^ On the other hand, imatinib showed a more favorable effect on treating fibrosis, since it prevented the proliferation and fibrosis of FAPs and had no impact on myoblast proliferation.^3^

The TGF-β inhibitor SB431542 was found to reduce rotator cuff muscle fibrosis and fatty infiltration by inducing FAP apoptosis.^121^ Batimastat, a broad matrix metalloproteinase inhibitor, can efficiently prevent adipogenesis of FAPs in muscle injury.^33^ Annexin A2 is another potential therapeutic target in muscle atrophy diseases. Hogarth et al demonstrated that accumulation of annexin A2 in the myofiber matrix could favor adipogenic induction of FAPs. It is the main reason contributing to fatty degeneration in limb girdle muscular dystrophy 2B, which is caused by mutations in dysferlin. Using batimastat can attenuate the fatty infiltration and degeneration of dysferlin-deficient muscle.^70^

Direct injection of FAPs may improve tissue repair. Recently, researchers injected beige-FAPs labeled with UCP-1^+^ Sca1^+^ Pdgfrα^+^ CD31^−^ CD45^−^ integrin α7^−^ into torn rotator cuff. They found that this transplantation could suppress fibrosis and fatty infiltration, thus promoting vascularization and shoulder recovery.^122^^,^^123^ In addition, adipose-resident FAPs can reinstate the regenerative function of muscle stem cells.^124^ In chronic obstructive pulmonary disease, CD34 expressed on FAPs was critical to maintain the normal function of muscles in hypoxic conditions. CD34 depletion can lead to a reduction of FAPs as well as impaired strength of muscles.^125^^,^^126^

Antioxidant compound tocotrienol (γ-tocotrienol, GT3) can reduce the production of reactive oxygen species in muscle stem cells of DMD mice, which is conducive to promoting the functional recovery and differentiation of muscle stem cells of DMD mice. At the same time, the application of GT3 can significantly reduce the percentage of Pdgfrα^+^ fibroblast adipo-progenitor cells in the tibialis anterior muscle of DMD mice, thus controlling the progress of fibrosis and relieving the pathological symptoms of DMD.^127^

## The role of Pdgfrα^+^ stromal cells in adipose

Adipose-resident Pdgfrα^+^ stromal cells reside in stromal vascular fraction. Increasing studies revealed that Pdgfrα^+^ stromal cells are preadipocytes, which are the major cells to differentiate into adipocytes.^128^, ^129^, ^130^, ^131^ In addition, they shared many similar properties with muscle-resident FAPs.

### Identification of Pdgfrα^+^ preadipocytes through biomarkers

Many Pdgfrα^+^ preadipocytes are found to be adjacent to blood vessels.^128^^,^^132^ Rodeheffer et al screened potential cells that possessed differentiative ability through fluorescence activated cell sorter. They selected several classic stem cell biomarkers, including Sca-1, CD29, and CD34, to identify potential adipogenic progenitors in stromal vascular fraction. The results showed both Lin^−^ CD34^+^ Sca-1^+^ CD29^+^ CD24^+^ and Lin^−^ CD34^+^ Sca-1^+^ CD29^+^ CD24^−^ subgroups could differentiate into adipocytes *in vitro*. Nevertheless, only Lin^−^ CD34^+^ Sca-1^+^ CD29^+^ CD24^+^ subgroup showed a potent capacity of adipogenesis *in vivo*, indicating that this subgroup was preadipocyte.^129^ Interestingly, the Pdgfrα^+^ CD24^+^ subgroup is the precursor of the CD24^−^ subgroup and accounts for a large population of adipogenic cells in the embryonic subcutaneous white adipose tissue. Meanwhile, the Pdgfrα^+^ CD24^−^ subgroup can differentiate into mature adipocytes after birth.^128^ Most Lin^−^ Pdgfrα^+^ cells are preadipocytes since they give rise to adipocytes.^131^ Meanwhile, another combination of biomarkers for adipose-resident FAPs was considered, including Lin^−^ Gp38^+^ Pdgfrα^+^.^133^ Together, Lin^−^ Sca-1^+^ Pdgfrα^+^ could be definitely used to identify Pdgfrα^+^ stromal cells in adipose tissues. In human adipose tissues, Lin^−^ CD34^+^ CD44^+^ Pdgfrα^+^ was used to sort out human adipose-derived FAPs (Table 1).^133^

### The dominant subpopulation of Pdgfrα^+^ preadipocytes

CD9 distinguished the different fates of adipose-derived Pdgfrα^+^ stromal cells. After HFD feeding, the Pdgfrα^+^ CD9^high^ subgroup showed a pro-fibrotic phenotype, while the Pdgfrα^+^ CD9^low^ subgroup was more likely to commit to adipogenic fate. In obesity, the Pdgfrα^+^ CD9^low^ subgroup was obviously diminished, and white adipose tissue exerted a pro-inflammatory status. Consistently, Pdgfrα^+^ preadipocytes derived from white adipose tissue in obese humans displayed pro-fibrotic features with high expression of α-SMA (Table 2).^133^

Interestingly, adipose-resident Pdgfrα^+^ stromal cells presented with an obvious heterogeneity. Using the single-cell RNA-seq technique, Schwalie et al identified that three major subgroups could be distinguished by surface markers, CD55 & IL13RA1 (group 1), VAP1 & Adam12 (group 2), and CD142 & ABCG1 (group 3), respectively.^130^ Notably, the CD142^+^ ABCG1^−^ subgroup was named as “Areg” because it displayed an inhibitory effect of adipogenesis through direct and paracrine manners. Rtp3, Spink2, FGF12, and Vit were involved in this adipogenic inhibition (Table 2).^130^ In another study, adipose-resident Pdgfrα^+^ stromal cells with the expression of dipeptidyl peptidase-4 showed a higher proliferation capacity and gave rise to committed ICAM1^+^ and CD142^+^ preadipocytes, indicating that the dipeptidyl peptidase-4-positive subgroup had a strong stemness.^134^ Interestingly, the aforementioned two studies presented a controversial function of the CD142^+^ subgroup in adipogenesis. This divergency is likely due to the heterogeneity in the CD142^+^ subgroup (Table 2).

### PDGF ligands regulating the adipogenesis of Pdgfrα^+^ preadipocytes

**Figure 5:**
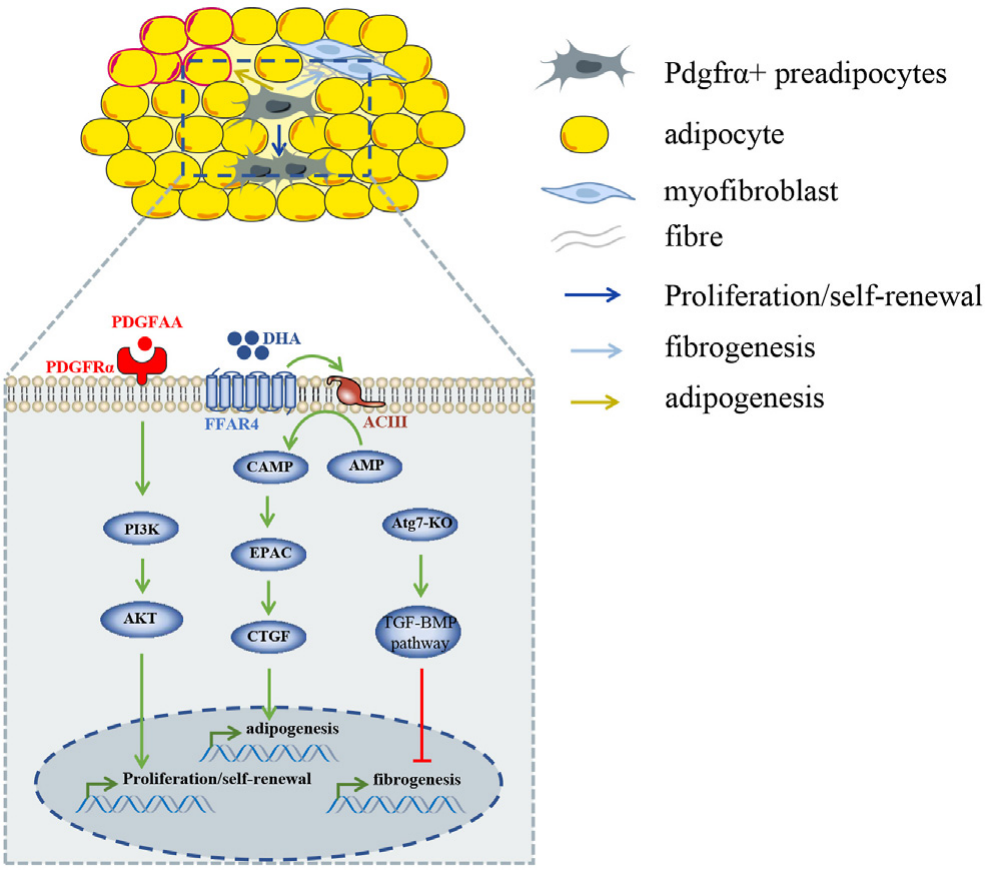
The signals that regulate Pdgfrα^+^ preadipocytes in adipose.

Similar to the muscles, PDGF ligands also regulate the biological process of adipose. It determines adipocyte–myofibroblast transition in white adipose tissues.^135^^,^^136^ In dermal adipose, the maintenance of adipose progenitors is controlled by the PDGFA/PI3K-AKT signaling pathway.^137^ In addition, it is well known that there are several types of adipocytes, including beige adipocytes and white adipocytes, the former of which can provide energy and thermogenesis. The Pdgfrα/Pdgfrβ signal determines the balance between beige adipocytes and white adipocytes. Pdgfrβ^+^ preadipocytes mainly contribute to white adipocytes and Pdgfrα^+^ preadipocytes can generate beige adipocytes (Fig. 5).^135^

### Cilia controlling the fate of Pdgfrα^+^ preadipocytes

Similar to muscle-resident FAPs, cilia play an important role in regulating Pdgfrα^+^ preadipocytes. The proliferation and adipogenesis of Pdgfrα^+^ preadipocytes and white adipose tissue expansion can be regulated by the cilia. It was reported that Pdgfrα^+^ preadipocytes which were located along blood vessels were ciliated before differentiating into mature adipocytes. The preadipocytes with cilium, including 3T3-L1 preadipocyte cell lineage and primary Pdgfrα^+^ preadipocytes, show sensitivity to proliferation. Consistently, loss of cilium contributes to a reduction of white adipose tissue. Further investigation has revealed that cilium is a sensor to varieties of signals. ω-3 fatty acids can accelerate the proliferation and differentiation of preadipocytes by mediating chromatin remodeling through FFAR4/cAMP/CTCF pathway. Finally, ω-3 fatty acids facilitate white adipose tissue expansion through enhancing Pdgfrα^+^ preadipocyte proliferation, thus improving insulin sensitivity and tissue inflammation.^138^ This result implies that adipogenesis of Pdgfrα^+^ preadipocytes in adipose is critical to regulating metabolic homeostasis (Fig. 5).

### Autophagy promoting fibrogenic capacity of Pdgfrα^+^ preadipocytes

HFD can also lead to fibrosis in adipose tissue by regulating the autophagic process in Pdgfrα^+^ preadipocytes. Autophagy related 7 was critical to the autophagy-induced fibrotic phenotype of Pdgfrα^+^ preadipocytes. Conditional autophagy related 7 knockout in Pdgfrα^+^ preadipocytes obviously attenuates ECM gene expression in visceral, subcutaneous, and epicardia fats, exerting a general effect on fibrosis caused by Pdgfrα^+^ preadipocytes.^139^ Actually, the role of autophagy in cellular function and fates of Pdgfrα^+^ preadipocytes are largely unknown, and further studies are needed (Fig. 5).

## The function of tendon-resident Pdgfrα^+^ progenitors

Tendons are dense connective tissues that connect muscles and bones, which are responsible for the mechanical load. After injury, tendons exhibit impaired healing potential with excessive scar formation.^46^ Tendon matrix continuity and longitudinal alignment are essential to tendon regeneration. However, the cellular components regulating tendon regeneration and degeneration remain elusive. Harvey et al recently identified three major groups of tendon-resident progenitors through single-cell sequencing based on expression levels of Pdgfrα and TPPP3 (Table 1). TPPP3^+^ Pdgfrα^+^ progenitors could differentiate into chondrocytes and osteocytes. TPPP3^−^ Sca-1^+^ Pdgfrα^+^ progenitors were tendon-derived FAPs, giving rise to adipocytes, chondrocytes, and osteocytes (Fig. 3). The third subgroup TPPP3^+^ Pdgfrα^−^ cells could only differentiate into chondrocytes. Then, they further investigated the functions of TPPP3^+^ Pdgfrα^+^ and TPPP3^−^ Pdgfrα^+^ subgroups and revealed that these two groups showed opposite functions in tendon repair. TPPP3^+^ Pdgfrα^+^ progenitors were tenogenically predisposed and contributed to tendon regeneration. However, tendon-derived FAPs contribute to scar formation in tendon injury. In normal conditions, FAPs often locate in the sheath. However, these FAPs will migrate into tissue after injury. It should be noted that the three subgroups co-existed in the same niche in the tendon. PDGFAA signaling modulates regeneration and fibrosis simultaneously.^46^ Thus, it is difficult to promote tendon regeneration and prevent scar formation simultaneously through regulating PDGFAA signaling. Further investigation should be performed to look for some therapeutic strategies that specifically target tendon-resident FAPs but not TPPP3^+^ tendon stem cells to attenuate scar formation.

## The Pdgfrα^+^ progenitors in ECM remodeling and repair of the cardiovascular system

Smooth muscle is essential to vascular repair, but the source of smooth muscle cells was unclear before. Although Sca-1^+^ cells were once identified to have the potential to differentiate into smooth muscle cells, the role of Sca-1^+^ cells in differentiating into smooth muscle cells *in vivo* was still unclear. To resolve this issue, Tang et al analyzed the subgroup of Sca-1^+^ cells in the femoral artery wall through single-cell RNA sequencing. Two subgroups were identified, which expressed Pdgfrα^+^ or Pdgfrβ^+^, respectively. Using lineage tracing mice, they found Sca-1^+^ cells did not contribute to the generation of smooth muscle cells in normal or slight injury models. However, when the artery suffered a severe injury, a mass of Sca-1^+^ cells-derived smooth muscle cells appeared in the injured site to repair the artery injury. Further investigation revealed that only the Pdgfrα^+^ subgroup participated in this process. In normal conditions, the Pdgfrα^+^ subgroup often locates out of the artery wall, but they will immigrate into the arterial wall if severe injury occurs. Mechanistically, the Pdgfrα^+^ subgroup can rapidly proliferate via activation of yes-associated protein 1 after severe injury. After eliminating this subgroup, the artery repair will be significantly impaired.^140^ Interestingly, they found that this group of cells negatively expressed CD45 and could form adipose tissue around the artery. Although this Pdgfrα^+^ subgroup was not named as FAPs in this study, the features were like FAPs (Table 2).

**Figure 6:**
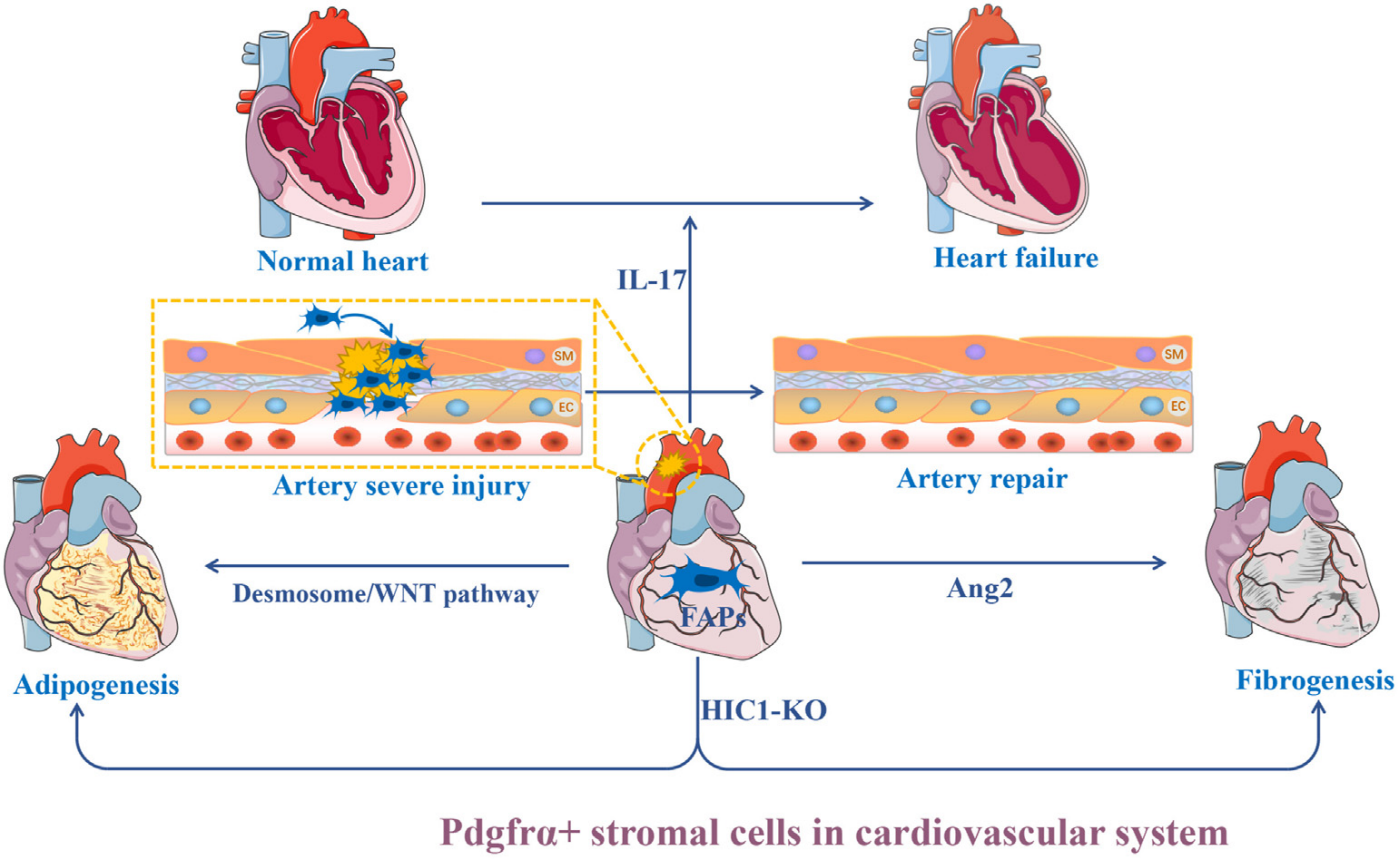
The role of Pdgfrα^+^ stromal cells in cardiovascular diseases.

In congenital and acquired cardiac diseases, fibrosis, and fatty infiltration are two typical pathological signs. Pdgfrα seems to be critical to pathological changes in cardiac development and diseases. Kim and colleagues identified Pdgfrα^+^ cardiac progenitors originated from multipotent germline stem cells. They found that Pdgfrα^+^ multipotent germline stem cells expressed more cardiogenic biomarkers than Pdgfrα^−^ multipotent germline stem cells. Transplantation of these Pdgfrα^+^ multipotent germline stem cells into rat myocardial infarction models can facilitate them to differentiate into functional cardiomyocytes and reduce fibrosis.^141^ Similarly, several studies distinguished subgroups of Sca-1^+^ cardiac stem/progenitor cells through Pdgfrα.^142^^,^^143^ Pdgfrα^+^ side population cells showed more exact cells enriched for cardiogenic transcripts.^143^ On the contrary, Chen et al found Lin^−^ CD29^+^ mEF-SK4^+^ Pdgfrα^+^ Sca-1^+^ periostin^+^ cardiac fibroblast subset highly shared common biomarkers with FAPs (Table 2). This group of cardiac fibroblasts contributed to heart failure in the presence of IL-17 by producing abundant granulocyte macrophage-colony stimulating factor.^144^ These studies implied that like in tendons, Pdgfrα^+^ progenitors might have distinct subgroups, which played different roles in heart repair (Fig. 6).

In 2016, Raffaella and colleagues identified cardiac FAPs encoding Pdgfrα and negatively expressing CD31, CD45, thymocyte antigen 1 (Thy-1), and discoidin domain receptor tyrosine kinase 2. These cells were bipotential. Collagen1α1 expressed broadly in these cells. Furthermore, a subset specifically expressed CEBP/α. Further investigation revealed adipogenic subsets of FAPs mainly expressed desmosome proteins and differentiated into adipocytes through a Wnt-dependent manner in arrhythmogenic cardiomyopathy.^145^ Cardiac FAPs are often set in epicardium.^145^^,^^146^ Quiescence-associated factor HIC1 was critical to the homeostasis of cardiac FAPs. Deletion of HIC1 in FAPs can lead to fibrofatty infiltration and cause major pathological features in arrhythmogenic cardiomyopathy.^147^ Contreras et al used PDGFRa-H2B:eGFP mice to isolate enhanced green fluorescent protein-labeled FAPs from the heart. They found TGF-β could inhibit the expression of Pdgfrα in heart-resident FAPs.^24^ In humans, cardiac FAPs located in epicardial layer can differentiate into myofibroblasts in the presence of angiotensin II. In pathological conditions, such as atrial fibrillation, subsets of cardiac FAPs can be reprogrammed towards a specific fate, leading to fibrofatty infiltration (Fig. 6).^148^

## The role of Pdgfrα^+^ stromal cells in lung injury and repair

In 2009, Lin^−^ Sca-1^+^ CD34^+^ Thy-1^+^ Pdgfrα^+^ mesenchymal cells were identified in the lung parenchyma (Table 1). These cells emerged during neonatal lung development and possessed fibroblastic, adipogenic, osteoblastic, and chondroblastic abilities, which were highly similar to muscle-resident FAPs.^149^

Pdgfrα^+^ fibroblasts showed obviously distinct features in lung injury and alveolar regeneration. Endale et al analyzed transcriptomic profiling and described the characteristics of Pdgfrα^+^ fibroblasts during lung development. They found Pdgfrα^+^ fibroblasts could immigrate from proximal bronchiolar at embryonic day 16.5–17.5 to distal alveolar location at postnatal day 5–28. Transcriptomic profiling showed that cell migration-associated genes were enriched at embryonic day 16.5. At embryonic day 18.5, these cells switched from smooth muscle cell phenotype to matrix-producing cells and lipofibroblasts, which exhibited FAP features. At postnatal day 7, several pathways were enriched in Pdgfrα^+^ fibroblasts, including ECM organization, angiogenesis, and epithelial development.^146^ These data revealed that these cells with features of FAPs in the lung migrated during development and exhibited distinct features in fibrosis and alveolarization.

Li et al found that Pdgfrα^+^ progenitors contributed to fibrosis induced by bleomycin in the lung through differentiating into myofibroblasts, but had little effect on hyperoxia-induced fibrosis. This indicated that lung-resident Pdgfrα^+^ progenitors had distinct lineage potential.^150^ Interestingly, when using lineage tracing, they found Pdgfrα^+^ cells could co-express early growth response and intercellular adhesion molecule-2, the biomarkers of endothelial cells.^150^ As we know, FAPs do not belong to endothelial cells. It was not sure whether FAPs could differentiate into endothelial cells in some special conditions, or whether Pdgfrα^+^ cells contained both endothelial cells and non-endothelial progenitors in the lungs. Zepp et al further investigated myofibroblast subgroups in the lungs. They identified three subgroups: Axin2^+^ Pdgfrα^+^, Axin2^+^, and Wnt2^+^.^151^ Axin2^+^ Pdgfrα^+^ mesenchymal cells located around alveolar type 2 progenitor cells form a mesenchymal alveolar niche, which promotes alveolar type 2 progenitor cell self-renewal and differentiation into alveolar type 1 progenitor cells. On the other hand, Axin2^+^ Pdgfrα^−^ cells may generate pathologically deleterious myofibroblasts after injury. This group was also the main source of airway smooth muscle cells. Notably, IL-6/STAT3 and FGF7 signals can promote alveolar type 2 progenitor cell self-renewal while BMP7 inhibited this process.^151^

The lung is a heavily attacked organ by SARS-COV-2 virus.^152^ Pulmonary fibrosis and impaired alveolar regeneration were two classic features of COVID-19.^153^, ^154^, ^155^ Inflammatory cytokine “storm” contributed to the lung injury. IL-6 was one of the most important cytokines that exacerbated lung injury. Mesenchymal stem cell therapy has been considered a promising choice for COVID-19.^156^^,^^157^ Considering the importance of Pdgfrα^+^ fibroblasts in lung injury and regeneration, it is worthy to explore their biological changes during infection of SAR-COV-2.

## Pdgfrα^+^ BMMSCs participating in bone regeneration

Bone marrow-derived mesenchymal stem cells (BMMSCs) were a group of MSCs located in bone and can be identified according to the markers Ter119^−^ CD45^−^ Sca-1^−^ Pdgfrα^+^ (Table 1). They highly express CD29, CD90, and CD44, and lowly express CD34.^158^ To our best knowledge, limited studies used these markers in their research on BMMSCs. One study showed that Sca-1^+^Pdgfrα^+^ BMMSCs could promote bone marrow regeneration.^159^ Another study exhibited that high-mobility group box 1 protein could recruit Pdgfrα^+^ BMMSCs to the peri-infarction site to promote re-vascularization and finally reduce fibrosis.^160^

## Pdgfrα^+^ PSCs in regulating the microenvironment of the pancreas

In fact, Pdgfrα^+^ stromal cells in pancreatic tissue have their tissue specific and widely recognized name, which is called pancreatic stellate cells (PSCs). PSCs can differentiate into fibroblasts and adipocytes. A recent article showed that the surface markers (Pdgfra^+^ CD31^−^ CD45^−^) of PSCs are similar to the muscle-resident FAPs.^161^ PSCs have two classical states: quiescent state and activated state. Quiescent PSCs are present by numerous prominent lipid droplets in the cytoplasm, α-SMA negative, and with limited proliferation and ECM production. When stimulated by endogenous and exogenous factors, PSCs activate into myofibroblast like phenotype with α-SMA positive and less or absent lipid droplets, showing significantly enhanced proliferation and ECM production capacity.^162^, ^163^, ^164^ Physiologically, activated PSCs have the potential to recover to a quiescent state. However, under the stimulation of some pathological conditions, such as chronic pancreatitis and pancreatic cancer, continuously activated PSCs with fibroblast like phenotype can promote the malignant progress of chronic pancreatitis and pancreatic cancer with their strong fibrogenesis ability, and become the center of pancreatic fibrosis. Here, we list some factors that regulate PSC activation and fibrosis in chronic pancreatitis.

### Cytokines stimulating fibrogenic capacity of PSCs

Persistent activation of PSCs by cytokines during acute pancreatitis such as TNF-α, IL-1, IL-6, and IL-10, may be a factor involved in the progression from acute pancreatitis to chronic pancreatic injury and fibrosis.^165^ Zheng M et al described a possible mechanism that IL-6 contributes to PSC activation and collagen І production through up-regulation of the TGF-beta1/Smad2/3 pathway.^166^ An impaired Rora/Nr1d1/Bmal1 loop, called the circadian stabilizing loop could result in the deficiency of pancreatic Bmal1, which accounted for the fibrogenic properties of PSCs in a clock-TGF signaling-IL-11/IL-11RA axis-dependent manner. Thus, a protective pancreatic clock had the potential against pancreatic fibrosis in chronic pancreatitis.^167^ What's more, Ng B et al put forward that anti-IL11RA could reduce pathologic (extracellular signal-regulated kinase, STAT, NF-kappa B) signaling in PSCs, and inhibit subsequent pancreatic atrophy and fibrosis.^168^ Recently, Xuguang Yang et al defined a subpopulation of PSCs, VLDLR^+^ PSCs, which were comparatively enriched in inflammatory responses, growth factor activity, and lipid metabolism-related pathways, and closely related to pancreatic fibrosis. In mechanism, increased intake of very low-density lipoprotein (VLDL) through VLDLR could promote the release of IL-33 from VLDLR^+^ PSCs via the LA-EBF2-IL-33 axis. Up-regulation of IL-33 aggravated the alcohol/pancreatic injury-induced pancreatitis fibrosis progression by activating the pancreatic ILC2s through its receptor suppression of tumorigenicity 2. On one hand, activated ILC2s recruited more type 2 immune cells, M2-like macrophages, and Th2 cells via IL-13/IL-4, which accounted for pancreatic fibrosis. On the other hand, activated ILC2s secreted IL-13/AMP/leukemia inhibitory factor, which resulted in fibroblast activation and proliferation of PSCs, eventually promoting fibrosis.^161^

### Autophagy regulating fibrogenesis of PSCs

Autophagy seems to promote PSC activation and subsequent fibrosis. A novel lncRNA named lnc-PFAR was demonstrated highly presented in mouse and human chronic pancreatitis tissues. lnc-PFAR enhanced PSC activation and pancreatic fibrosis through trigging a miR-141-RB1CC1 (RB1-inducible coiled-coil 1) axis-dependent-autophagy.^169^ Therefore, inhibition of autophagy may become one of the targets for the treatment of fibrosis. A study pointed out that, the knockdown of RB1CC1 could block autophagy-dependent activation of PSCs and impaired pancreatic fibrosis in chronic pancreatitis.^170^ Similarly, milk fat globule epidermal growth factor 8 appeared to alleviate pancreatic fibrosis via inhibiting lysosome associated membrane protein type 2A in chaperone-mediated autophagy and subsequent activation of PSCs.^171^ Also, vitamin E derivatives tocotrienols selectively trigger the autophagy of inactivated PSCs by targeting mitochondrial permeability transition pore and ameliorated fibrogenesis associated with chronic pancreatitis.^172^

### Unhealthy lifestyle promoting pancreatic fibrosis through activation of PSCs

Alcohol could accelerate the progression of pancreatic fibrosis. Repeated lipopolysaccharide resulted in significantly greater pancreatic fibrosis in alcohol-fed rats compared with rats fed the control diet without alcohol. Notably, PSCs were activated by lipopolysaccharide. Lipopolysaccharide plus alcohol exerted a synergistic effect on PSC activation and pancreatic fibrosis.^173^ Continued alcohol administration prevented PSC apoptosis and perpetuated pancreatic injury/fibrosis. Withdrawal of alcohol led to increased PSC apoptosis and resolution of pancreatic lesions including fibrosis.^174^ In addition, smoking contributes to PSC fibrosis. Ah receptor ligands found in cigarette smoke increased the severity of pancreatic fibrosis. In mechanism, Ah receptor ligands promoted the release of IL-22 from pancreatic T cells, which further activated the fibrogenic potential through IL22RA1 in PSCs.^175^ Meanwhile, Li Z et al indicated that nicotine facilitates pancreatic fibrosis by promoting the activation of PSCs via the alpha7nAChR-mediated JAK2/STAT3 signaling pathway.^176^

### Other factors influencing the fate of PSCs

It has been reported that intracellular oxidation levels can regulate fibrosis. Nicotinamide adenine dinucleotide phosphate oxidase 1-derived reactive oxygen species in PSCs accelerated the fibrotic process of chronic pancreatitis by activating the downstream pathways AKT and NF-kB, raising matrix metalloproteinase 9 and Twist, and producing alpha-smooth muscle actin and collagen I and III.^177^ The antioxidant, mitoquinone (MitoQ) inhibited PSC activation as well as the transition of the profibrogenic phenotypes by balancing the levels of free radicals and the intracellular antioxidant system, meaning that MitoQ is a potential candidate treatment for chronic pancreatitis.^178^ Non-coding RNA also participates in the regulation of the fate of PSCs. miR-301a is highly expressed in activated PSCs in mice, sustaining tissue fibrosis in caerulein-induced chronic pancreatitis via Tsc1/mTOR and Gadd45g/STAT3.^179^ Acinar cell-derived exosomal miR-130a-3p promoted PSC activation and collagen formation through targeting of stellate cellular PPARγ. Thus, the knockdown of miR-130a-3p significantly provided a potential new target for the treatment of chronic pancreatic fibrosis.^180^

## Conclusions

Pdgfrα^+^ stromal cells participate in degeneration and regeneration in varieties of tissues and organs. They share similarities and have unique and important features. Pdgfrα^+^ stromal cells in most tissues play a “double-edged sword" effect. This feature indicates that depletion of Pdgfrα^+^ stromal cells would not be a proper therapeutic strategy. Moreover, Pdgfrα^+^ stromal cells are multipotent mesenchymal cells. Noticeably, Pdgfrα^+^ stromal cells are susceptible to surroundings. They are easily modified by various cytokines and cells.

Pdgfrα^+^ stromal cells lead to fibro-fatty infiltration in most organs and tissues. In our opinion, adipocyte accumulation and fibrosis in degenerative diseases might be a passive defense to cope with unlimited muscle atrophy. In addition, Pdgfrα^+^ stromal cell-derived adipocytes show the features of “good” adipocytes, *i.e.*, beige adipose. The role of proper adipogenesis in muscle degeneration should be further investigated.

Senescence and apoptosis are critical to maintain tissue functions. Rapid reduction of Pdgfrα^+^ stromal cells is helpful to tissue regeneration, to avoid forming excessive fibrosis and adipose. Trp53 is a key regulator for senescence and apoptosis in muscle-resident Pdgfrα^+^ stromal cells. Overexpression of Trp53 can restrict the proliferation of muscle-resident Pdgfrα^+^ stromal cells and reduce abnormal fatty infiltration and fibrosis. Moreover, they down-regulate the expression of CD47 and PD-L1 to facilitate apoptotic Pdgfrα^+^ stromal cells being phagocytosed by macrophages.

Pdgfrα^+^ stromal cells in different tissues can be subdivided into several subgroups. Interestingly, the Osr1^+^ subgroup and CD142^+^ ABCG1^+^ population have been identified in muscle-resident FAPs and Pdgfrα^+^ preadipocytes, respectively. The Osr1^+^ subgroup can give rise to Pdgfrα^+^ stromal cells in limb development and is the main subgroup in the late stage of muscle repair in adults. The evidence implies that the Osr1^+^ subgroup may be a more primitive Pdgfrα^+^ stromal cells. Adipose-derived CD142^+^ ABCG1^+^ Pdgfrα^+^ preadipocytes are also named as “Areg”, which inhibit the adipogenesis of preadipocytes. However, sole CD142 cannot indicate Areg. The identification of Areg demonstrates that huge heterogeneity exists in subgroups of Pdgfrα^+^ preadipocytes. It is interesting to explore whether Areg transplantation or its secreta can efficiently treat fatty degeneration in degenerative diseases.

Cardiac FAPs are currently considered to predominantly contribute to fatty infiltration and fibrosis in cardiac diseases. In addition, artery-derived Pdgfrα^+^ stromal cells can differentiate into smooth muscle cells in the injured artery. Thus, it is interesting to explore whether cardiac FAPs can turn into myocardial cells in some conditions.

Taken together, studies about Pdgfrα^+^ stromal cells have increased dramatically in the past several years. However, the roles of Pdgfrα^+^ stromal cells in regeneration and degeneration are still far from clear. Pdgfrα^+^ stromal cells distribute in most tissues and organs because they are located in interstitial mesenchyme and adjacent to vessels. Further studies on Pdgfrα^+^ stromal cells in different locations might shed light on the management of associated diseases.

## Ethics declaration

This article does not contain any studies with human or animal subjects performed by any of the authors.

## Author contributions

X.K., X.Q., S.F., and H.M. wrote the manuscript with the assistance of K.Z. K.Z. and H.Z. performed the drawing work.

## Conflict of interests

The authors declare no competing interests.

## Funding

This work was supported by grants from the 10.13039/501100001809National Natural Science Foundation of China (No. 82173134 to H.M.), the Sichuan Science and Technology Program (China) (No. 2023ZYD0061 to X.K.), the funding of Jinfeng Laboratory (Chongqing, China), 10.13039/501100002858China Postdoctoral Science Foundation (No. 2020M673648 to X.K.), 10.13039/501100009976Chongqing Postdoctoral Science Special Foundation (China) (to X.K.), and Chongqing Science and Technology Bureau (China) (No. CSTB2022TIAD-KPX0168 to X.Q.).
